# Biochemistry of malaria parasite infected red blood cells by X-ray microscopy

**DOI:** 10.1038/s41598-017-00921-2

**Published:** 2017-04-11

**Authors:** S. Kapishnikov, L. Leiserowitz, Y. Yang, P. Cloetens, E. Pereiro, F. Awamu Ndonglack, K. Matuschewski, J. Als-Nielsen

**Affiliations:** 1 Niels Bohr Institute, University of Copenhagen, Universitetsparken 5, DK-2100 Copenhagen Ø, Denmark; 2 Department of Materials and Interfaces, Weizmann Institute of Science, Rehovot, 76100 Israel; 3 European Synchrotron Radiation Facility (ESRF), 71 avenue des Martyrs, 38000 Grenoble, France; 4 ALBA Synchrotron Light Source, MISTRAL Beamline-Experiments Division, 08290 Cerdanyola del Valles, Barcelona, Spain; 5 Molecular Parasitology, Institute of Biology, Humboldt University, Philippstr. 13, 10115 Berlin, Germany

## Abstract

Red blood cells infected by the malaria parasite *Plasmodium falciparum* are correlatively imaged by tomography using soft X-rays as well as by scanning hard nano-X-ray beam to obtain fluorescence maps of various elements such as S and Fe. In this way one can deduce the amount of Fe bound either in hemoglobin or in hemozoin crystals in the digestive vacuole of the malaria parasite as well as determine the hemoglobin concentrations in the cytosols of the red blood cell and of the parasite. Fluorescence map of K shows that in the parasite’s schizont stage the K concentration in the red blood cell cytosol is diminished by a factor of seven relative to a pristine red blood cell but the total amount of K in the infected red blood cell is the same as in the pristine red blood cell.

## Introduction

Part of the complex life cycle of the malaria parasite *Plasmodium Falciparum* involves invasion and occupation of a red blood cell. There it catabolizes hemoglobin in its digestive vacuole in order to grow and asexually multiply into a new generation of parasites that can subsequently invade new healthy red blood cells. A byproduct of the degraded hemoglobin is heme, a porphyrin ring with a central iron (Fe) atom, which is precipitated into hemozoin crystals consisting of dimers of the heme molecule^1–4^. Monomers of heme in solution in the digestive vacuole cytosol would be toxic to the parasite as they are highly reactive, but in form of hemozoin crystals the heme is harmless^4^. Obvious targets for drug design would be impedement either of hemozoin crystal growth^5^, or of the protein assisted dimerization of heme^6, 7^.

**Figure 1:**
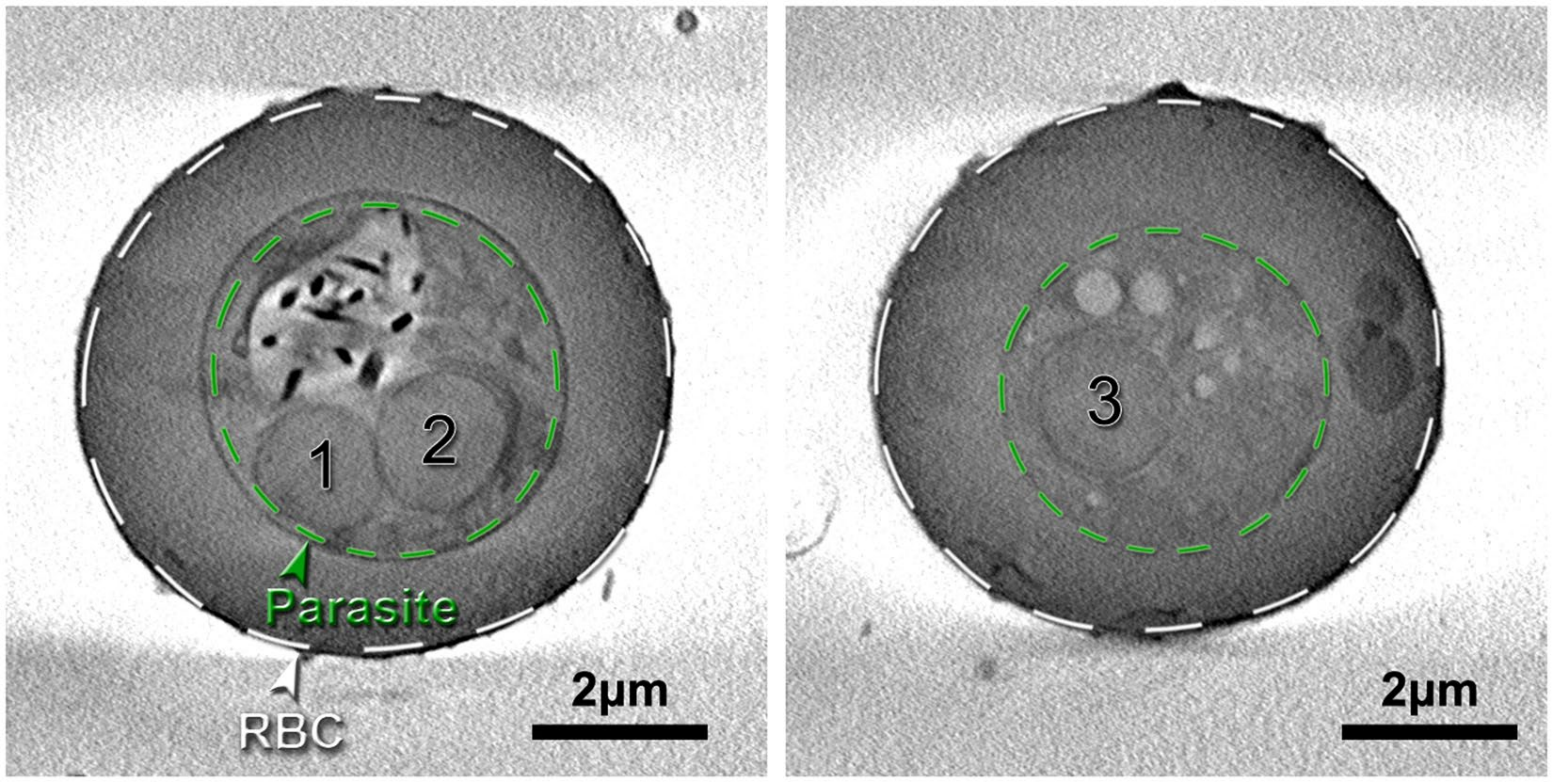
Two 226 nm thick slices 1356 nm apart of the reconstructed infected red blood cell (RBC) data from soft X-ray absorption. The left panel is a slice cutting through Hz crystals and two nuclei marked 1 and 2 are clearly visible. The right panel is a slice beyond the Hz crystals but shows a third nucleus. The white dashed line delineates the red blood cell, whereas the green dashed line marks the parasite slightly inside its membrane. Based on the volumes and the appearance of three nuclei we assessed the age of the parasite to be 38–44 hours after invasion^10^. The orientation of these slices was chosen because it provides the highest resolution in cryo-SXT image although it differs slightly from the projection angle of the infected RBC shown in the following figures.

**Figure 2:**
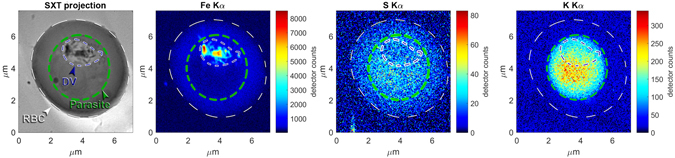
Maps of the red blood cell (RBC) infected with the malaria parasite in the schizont stage. The greyscale map is a soft X-ray radiograph at the same orientation of the beam relative to the sample as the X-ray fluorescence maps shown in color. Different compartments derived from the cryo-SXT are in the fluorescent maps outlined in white (entire cell), green (parasite), blue (digestive vacuole).

**Figure 3:**
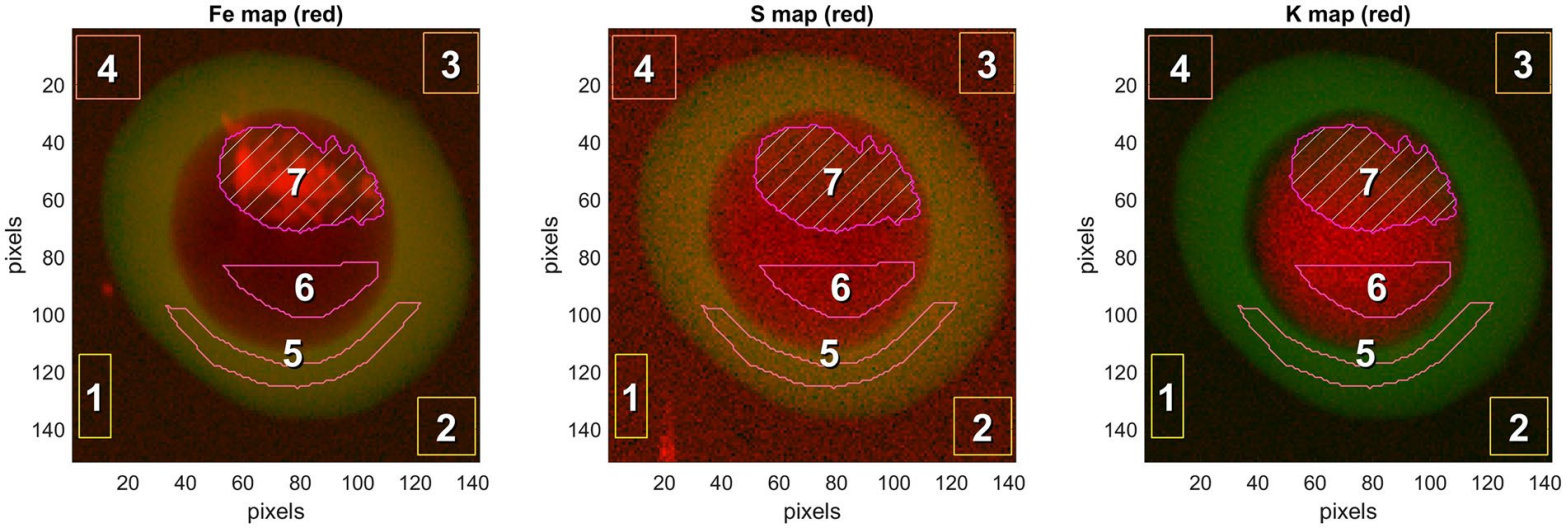
Left: Overlay of Fe fluorescence (log scale and red) with cryo-SXT segmentation (green) of the red blood cell (RBC) cytosol, the latter being the difference of the entire cell and the parasite. Overlay of green and red appears as brown which is then the Fe remaining in the RBC cytosol. The cross-hatched region denotes the DV and the bright red color therein shows the hemozoin crystals. Most of the Fe is either in the RBC cytosol in the form of hemoglobin, or in the DV in the form of hemozoin crystals. Middle: Overlay of S fluorescence (red) with cryo-SXT segmentaion (green) of the RBC cytosol. The ratio of S:Fe is much larger than 3:1 so the S in the parasite cytosol must predominantly be bound in other molecules than hemoglobin. Right: Overlay of the K fluorescence (red) with cryo-SXT segmentation (green) of the RBC cytosol. 80 per cent of the K originally in the RBC cytosol is at this stage inside the parasite membrane. The masks labelled 1 to 7 are used for determining concentrations in the corresponding volumes.

We have developed a methodology to obtain *in*-*situ* images of the parasite that may help in intelligent design of such drugs. Infected red blood cells derived from a culture are cryo-freezed to preserve their hydrated native structure^8, 9^, so the sample to be studied is like an insect in amber. The freezing is done so fast that the ice is vitrified into an amorphous structure rather than into ice crystals. An infected red blood cell is then imaged using computed tomography (CT) by the same principle as in medical CT scans, just at much higher resolution and much smaller X-ray beams that can be obtained from synchrotron X-ray sources. From the data set one can then generate slices of the three dimensional structure at any arbitrary orientation or thickness. Since these slices are extracted with software they are called virtual slices, in contrast to the real slices used in electron microscopy. An example of two virtual slices is shown in Figure 1. The left panel is a slice through the parasite showing the digestive vacuole as the light grey area with distinct hemozoin crystals (black) and two nuclei. The right panel is a slice behind the digestive vacuole showing a third nucleus. The number of nuclei can be used as a signature of the time elapsed since the parasite invasion into a red blood cell^10^. In this case three nuclei indicate a time lapse of 38–44 hours. The contrast in these images is obtained by using an X-ray energy (520 eV) in the so-called water window between the absorption edges of carbon and oxygen. X-rays in this energy region are termed soft X-rays. The same infected red blood cell is subsequently scanned through a hard X-ray beam (17 keV) yielding X-ray fluorescence (XF) maps from excited atoms such as iron, sulfur and potassium, see Figure 2. The hemozoin crystals stand out clearly in the Fe map, but there is also signal from the Fe bound in hemoglobin in the red blood cell cytosol. The CT data can be used to generate an absorption projection which can be thought of as a stack of slices, see left panel in Figure 2. The two kinds of images, absorption projection and fluorescence maps, are overlaid in Figure 3. Of particular interest is the fluorescent yield of sulfur relative to that of iron. The hemoglobin molecule has 12 sulfur atoms and 4 Fe atoms, so if Fe is bound in hemoglobin the fluorescent map would reflect this ratio, whereas a map of a region only containing hemozoin crystals has no S signal but a large Fe signal. Finally a map with sulfur signal but no Fe signal would indicate presence of some protein molecules different from hemoglobin. The potassium signal is also of biological interest^11^. Figures 2 and 3 show that the RBC cytosol is depleted in K at this schizont stage^10^ whereas the parasite has a large concentration of potassium. In the following sections we shall describe the two imaging methods in more detail, and summarize quantitative results in Table 1.

**Table 1:** X-ray fluorescence results for the elements S, K and Fe.

	S	K	Fe
1 Absorption cross section, ${\sigma }_{a}^{Z}$ (barn)	541	1146	3642
2 Fluorescent yield, *η* ^*Z*^ (per cent)	8.04	14.3	35.4
3 Detector efficiency, *ε* ^*Z*^ (per cent)	82.8	93.8	99.1
4 Attenuation length in ice, *a* ^*Z*^ (m)	24	68	497
5 Count rate in mask 2	0.167 · 10^4^	0.772 · 10^4^	2.63 · 10^4^
6 Concentration of atoms outside RBC, ${\rho }_{O}^{Z}$ (atoms/μm^3^)	1.90 · 10^6^	1.73 · 10^6^	0.653 · 10^6^
7 Concentration of atoms in RBC, ${\rho }_{RBCcyto}^{Z}$ (atoms/μm^3^)	38.3 · 10^6^	21.9 · 10^6^	12.1 · 10^6^
8 Number of atoms in RBC	316 · 10^7^	180 · 10^7^	99.7 · 10^7^
9 Concentration of atoms outside iRBC, ${\rho }_{O}^{Z}$ (atoms/μm^3^)	2.13 · 10^6^	1.19 · 10^6^	0.673 · 10^6^
10 Concentration of atoms in iRBC cytosol, ${\rho }_{RBCcyto}^{Z}$ (atoms/μm^3^)	17.8 · 10^6^	3.03 · 10^6^	7.24 · 10^6^
11 Concentration of atoms in parasite cytosol, ${\rho }_{Pcyto}^{Z}$ (atoms/μm^3^)	26.6 · 10^6^	50.5 · 10^6^	0.634 · 10^6^
12 Concentration of atoms in DV, ${\rho }_{DV}^{Z}$ (atoms/μm^3^)	26.0 · 10^6^	46.8 · 10^6^	99.2 · 10^6^
13 Number of atoms in iRBC cytosol	173 · 10^7^	29.4 · 10^7^	70.3 · 10^7^
14 Number of atoms in parasite cytosol	70.2 · 10^7^	133 · 10^7^	1.67 · 10^7^
15 Number of atoms in DV	10.4 · 10^7^	18.7 · 10^7^	39.7 · 10^7^
16 Number of atoms in all compartments	253 · 10^7^	181 · 10^7^	112 · 10^7^

Line 1–4 the element-dependence of quantities in eq. 1.

Line 5 integrated count rate within the mask 2 of pristine RBC, ${\sum }_{(ij)\in 2}{C}^{Z}(i,j)$.

Line 6–7 concentrations in pristine red blood cell (RBC).

Line 8 absolute number of atoms in pristine RBC.

Line 9–12 concentrations in different compartments of infected red blood cell (iRBC).

Line 12–16 absolute number of atoms in different compartments of iRBC.

## Correlative X-ray imaging

**Figure 4:**
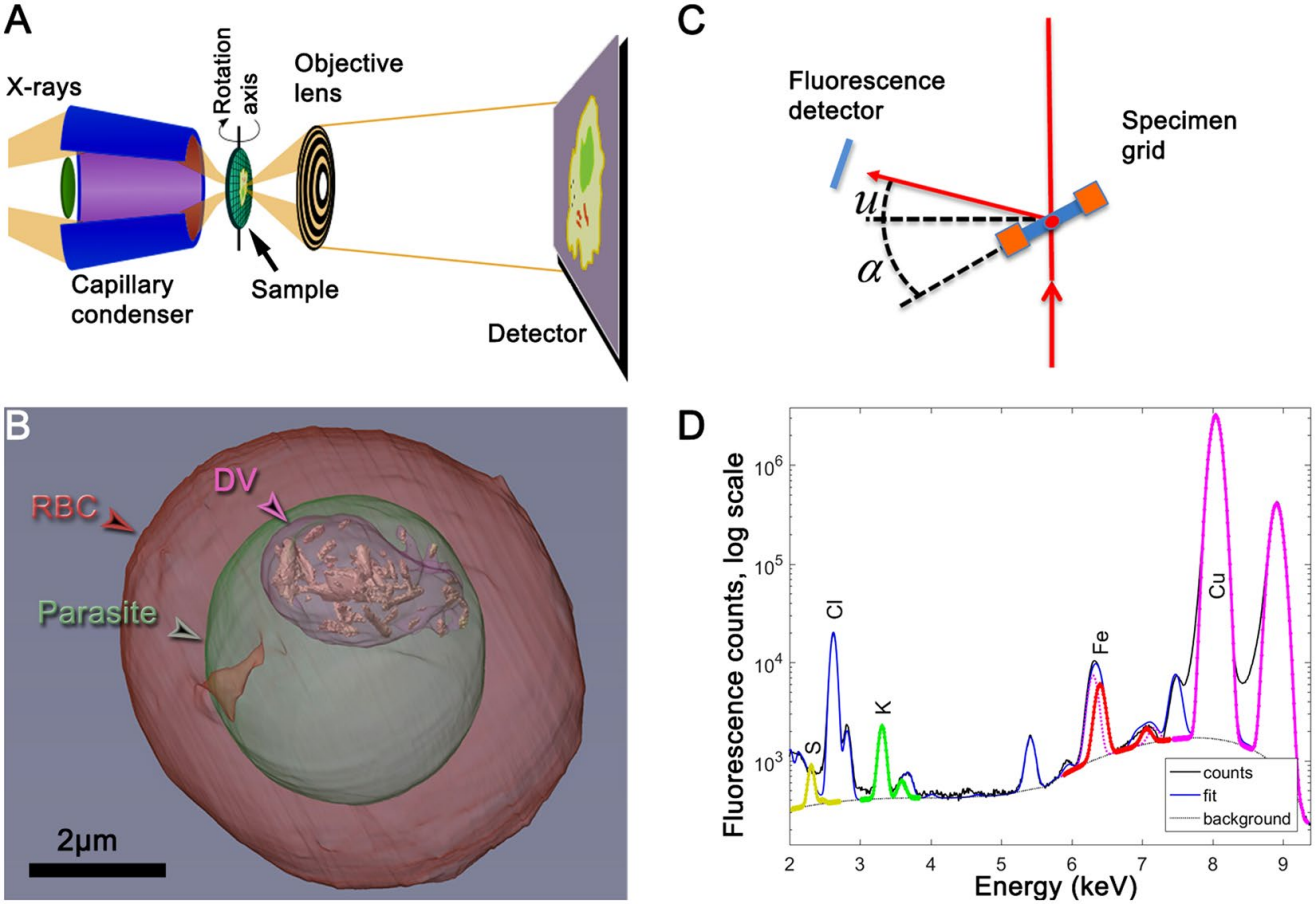
(**A**) Principles of the soft X-ray microscope, operated at 520 eV in the so-called “water window” range^19^. Soft X-ray projection images are obtained in one-degree consecutive steps for an angular range up to 131 degrees. (**B**) The images from all angles are combined to reconstruct a tomogram where different compartments of the infected red blood cell are segmented and given color for clarity. (**C**) X-ray fluorescence imaging. The sample is raster scanned in both dimensions perpendicular to the incident 17.05 keV beam, and the fluorescent X-rays are detected at the direction given by the angles *u* and *α*. The angle *u* was fixed at 17.8° whereas the angle between the incident X-ray beam and the normal to the grid plane, *α*, can be varied. With *α* > 20° the fluorescent rays are not intercepted by the Cu grid and will all enter the detector. (**D**) Fluorescent spectrum summed over all pixels defined by a mask. The large Cu signal is due to stray fluorescence from the Cu grid. The fit is derived by PyMCA program provided by ESRF.

The principle of cryo soft X-ray tomography (cryo-SXT) is depicted in the left hand side of Figure 4. The red blood cell is imaged by absorption contrast in an X-ray microscope at a soft X-ray energy in the so-called “water window”, *cf*. Figure 4A. In this energy interval, which is between the absorption edges of carbon (C) and oxygen (O), water is essentially transparent but there is good contrast on C and N, and thereby on lipid and protein molecules. Projection images obtained at many incident angles are combined to a tomogram, where one can distinguish different compartments in the cell, such as the parasite and the digestive vacuole within it. These compartments are given artificial wallcolors for clarity in Figure 4B. The first cryo-SXT from *Plasmodium falciparum* infected red blood cells were obtained by Hanssen *et al*.^12^ and Kapishnikov *et al*.^13, 14^. The cryo vitrification of the cell will, in addition to preserving native structures, also drastically diminish radiation damage^15^. The sample is situated in a specimen grid used for electron microscopy so it can be transferred and precisely located at a different X-ray beamline designed for the second method: cryo X-ray fluorescence (XF) imaging.

**Figure 5:**
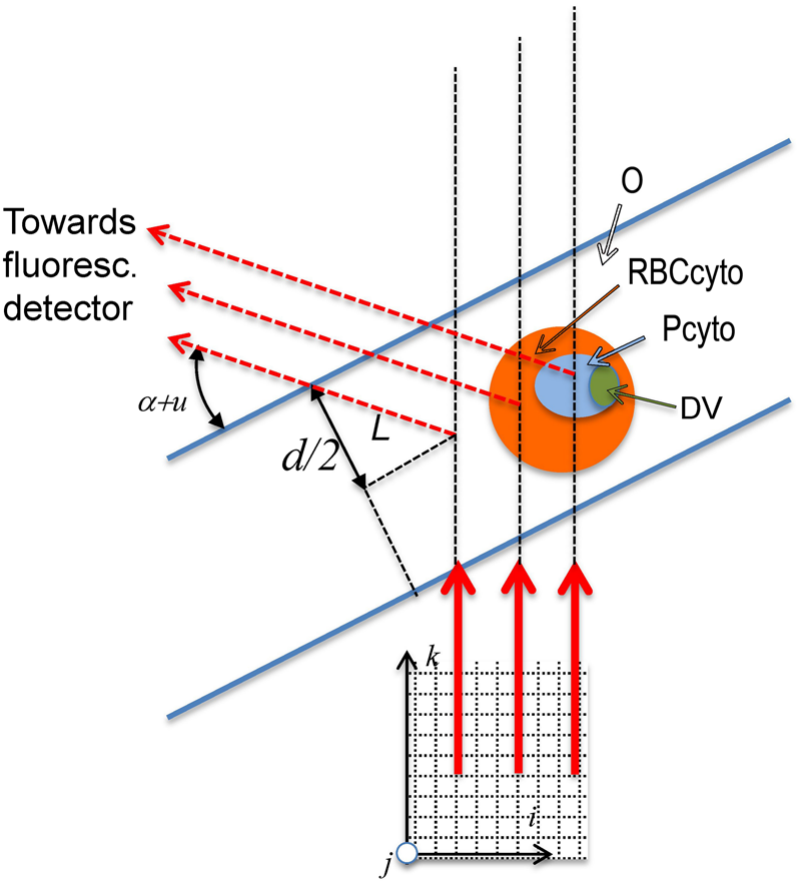
Principle of X-ray fluorescence imaging illustrated by a malaria parasite infected red blood cell (RBC). The RBC cytosol (RBCcyto), parasite (Pcyto) and inside the parasite the digestive vacuole (DV) are shown in orange, blue and green respectively. The vitrified ice of thickness *d* is between the two blue lines at angle *π*/2 − *α* relative to the beam. The sample is partitioned into voxels given by the integer coordinates $(i,j,k)$ with *k* being in the direction of the incident X-ray beam. Three positions of *i* in the scan of the sample are shown by the red arrows denoting the X-ray beam: one traverses only the vitrified ice, the next only the cytosol of the infected RBC, and the third traverses also the parasite cytosol. The relevant path lengths in the different compartments are derived from the cryo soft X-ray tomography. The fluorescent X-rays are shown by the dashed arrows.

The principle of X-ray fluorescence mapping of the density distribution of different elements throughout the cell is depicted in the right part of Figure 4. The hard X-ray beam will upon absorption give rise to X-ray fluorescent radiation such as *K*_*α*_ − *K*_*β*_ doublets from Fe, S and K atoms in the red blood cell. A map of the location of different atoms in the red blood cell can then be obtained by raster scanning the sample across the X-ray beam. The map shown in Figure 5 was done with a raster scan step of 0.050 micrometers covering an area of 7 × 7 μm^2^. This comprised about 20000 beam positions. For each beam position an entire fluorescent spectrum, spanning typically 1000 energies, was recorded. The complete scan was therefore a measurement of about two million data points. The addition of spectra within the raster area depicted “2” in Figure 3 is shown in Figure 4D.

## Analysis of fluorescent maps

For a given element, denoted by superscript *Z*, the count rate is proportional to the incident photon rate (*I*_0_), the solid angle subtended by the detector (ΔΩ), the absorption cross section (${\sigma }_{a}^{Z}$), the fluorescent yield (*η* ^*Z*^), the detector efficiency (*ε* ^*Z*^), and finally the product of number density of atoms (*ρ* ^*Z*^) and the traversed thickness *t*:

1 $${C}^{Z}={I}_{0}\cdot {\sigma }_{a}^{Z}\cdot {\eta }^{Z}\cdot ({\rm{\Delta }}{\rm{\Omega }}/4\pi )\cdot {\varepsilon }^{Z}\cdot {e}^{-L/{a}^{Z}}\cdot {\rho }^{Z}t$$

Although the fluorescent X-ray path *L* in the sample is only a few micrometers self-absorption must be corrected for by the exponential factor ${e}^{-L/{a}^{Z}}$ where *a* ^*Z*^ is the element specific attenuation length.

The simplest example is when the beam is scanned over an area of the vitrified ice outside the cell. Ideally, there should then be no fluorescence from atoms heavier than oxygen. However, the buffer solution used in the sample preparation as well as contents of a fraction of red blood cells ruptured during the vitrifying process at high pressure imply a fluorescence spectrum from many elements as shown in the lower right part of Figure 4D. *K*_*α*_ − *K*_*β*_ doublets from S, Cl, K, and Fe stands out clearly, but the spectrum is dominated by fluorescence from Cu. Although the beam area is much smaller than the Cu grid mesh of the sample support, stray radiation is the cause of this unwanted contamination, which furthermore implies so-called Cu escape doublets 1.4 keV below the *K*_*α*_ − *K*_*β*_ energies for Cu. However, by analyzing the spectrum with PyMCA software package^16^, the true fluorescent count rate from each element can be obtained, and from equation (1) one can determine the number density *ρ* ^*Z*^ of the elements.

When the beam is scanned through the RBC the formula becomes more complicated. We define the position of the sample relative to the beam by the indices (*i*, *j*), *cf*. Figure 5, and the different compartments that the (*i*, *j*) beam is traversing by a subscript *C*, where *C* can stand for outside the cell (*O*), RBC cytosol (*RBC*_*cyto*_), parasite cytosol (*P*_*cyto*_), or digestive vacuole (*DV*). The traversed path length in compartment *C* now depends on the beam position (*i*, *j*) and is denoted *t*_*C*_(*i*, *j*). It is determined from the cryo-SXT, so the only unknowns are the atomic densities in the different compartments, ${\rho }_{C}^{Z}$.

2 $${C}^{Z}(i,j)={I}_{0}{\sigma }_{a}^{Z}{\eta }^{Z}({\rm{\Delta }}{\rm{\Omega }}/4\pi ){\varepsilon }^{Z}{e}^{-L/{a}^{Z}}\cdot [\sum _{C}{\rho }_{C}^{Z}\cdot {t}_{C}(i,j)+{\rho }_{O}^{Z}\cdot ({t}_{O}-\sum _{C}{t}_{C}(i,j))]$$

In this equation the last term in the squared parentheses shows explicitly the contribution from the ice upstream and downstream of the RBC. One can define a mask, *M*, in the $(i,j)$ plane and sum the count rate over the *N*_*M*_ pixels in this mask to be ${C}_{M}^{Z}$. The cryo-SXT data determine which part of the $(i,j)$ path is within a given compartment *C*, and the sum of traversed paths within *C* is written as 〈*t*_*C*_〉 · *N*_*M*_, i.e. ${\sum }_{(ij)\in M}{t}_{C}(i,j)={N}_{M}\cdot \langle {t}_{C}\rangle$. By segmentation of the cryo-SXT data one can determine 〈*t*_*C*_〉 · *N*_*M*_. It follows from equation (2) by summation over $(i,j)\in M$ within the mask that

3 $${C}_{M}^{Z}={I}_{0}{\sigma }_{a}^{Z}{\eta }^{Z}({\rm{\Delta }}{\rm{\Omega }}/4\pi ){\varepsilon }^{Z}{e}^{-L/{a}^{Z}}\cdot {N}_{M}\cdot \sum _{C}[{\rho }_{C}^{Z}\langle {t}_{C}\rangle +{\rho }_{O}^{Z}\cdot ({t}_{O}-\langle {t}_{C}\rangle )]$$

with the densities ${\rho }_{C}^{Z}$ being the only unknown. Since the cryo-SXT also determines the volume of each compartment, one can derive the total number of atomic species in each compartment.

## Pristine red blood cell

An uninfected red blood cell (RBC) may serve as a convenient reference for the methodology of fluorescent imaging since the cell incorporates a total of about 2.7 *·* 10^8^ hemoglobin molecules^17^, each of which contains 4 Fe atoms and 12S atoms. Soft X-ray projection radiographs at different angles show that the cell accidentally is almost spherical.

**Figure 6:**
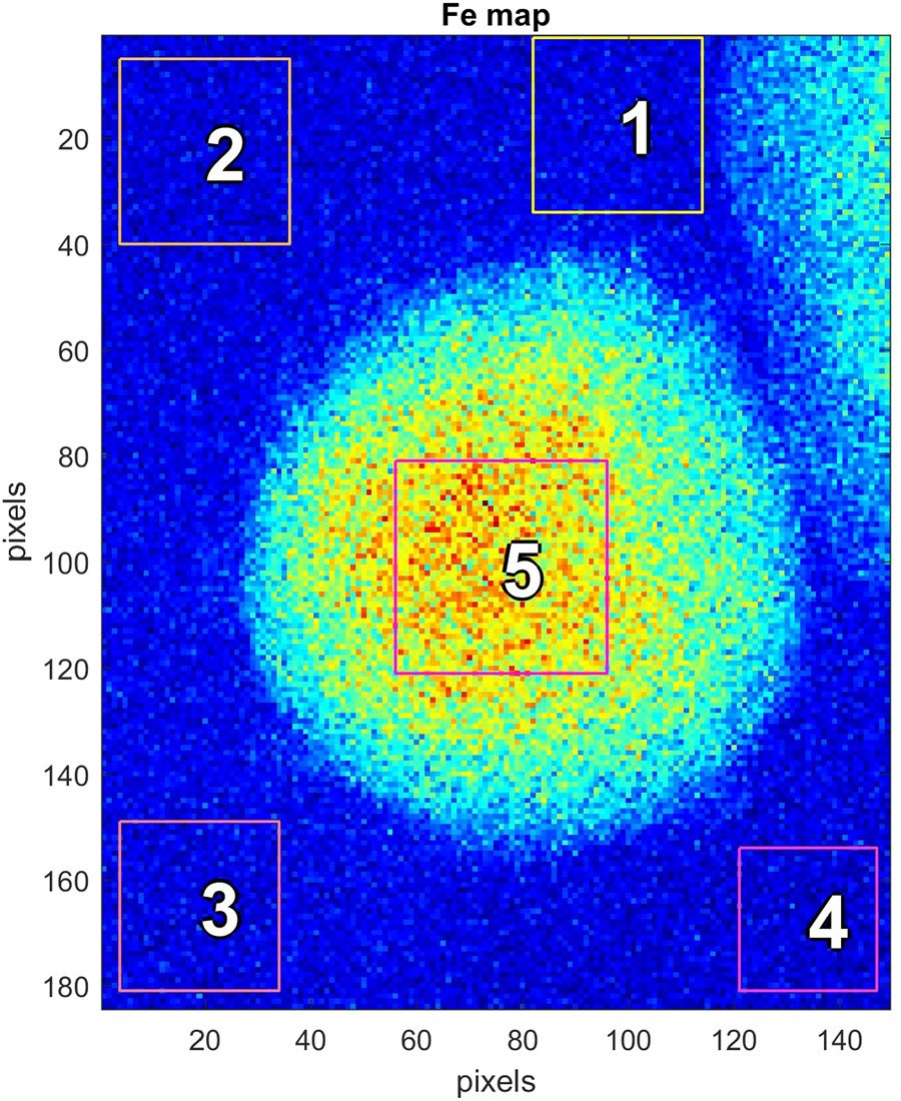
Fe map of a pristine red blood cell (RBC). One pixel is 50 nm. The rectangles are masks of raster areas from which X-ray fluorescence spectra are derived. Within masks 1–4 the scanning X-ray beam has illuminated the volumes outside the RBC with a traversed thickness of about 19 μm of ice. In mask 5, according to the tomogram of this sample, a 5.4 μm thickness of the RBC plus 13.6 μm of ice upstream and downstream have been illuminated.

In Figure 6, the map of Fe content is shown with a central quadratic mask and four masks outside the cell. By inspection it was verified that the count rate per pixel was the same in all four background masks. The spectrum for background mask 2 was shown already in Figure 4D. Using equation 1 the densities *ρ* ^*Z*^ of S, K, and Fe in the vitrified state can be determined, since the traversed path length *t* is the sample thickness *d* divided by cos(*α*). The element specific quantities labeled with superscript Z in equation 1 are given in Table 1, rows 1–4. The summed count rate in mask 2 for S, K and Fe is shown in row 5, while the concentrations and the absolute numbers of these atoms in various cellular compartments are shown in rows 6–16 of the same Table.

**Figure 7:**
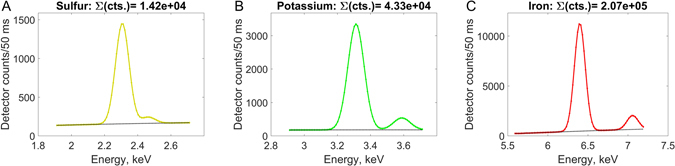
X-ray fluorescence spectra of (**A**) sulfur, (**B**) potassium and (**C**) iron from the projected area labeled 5 in Figure 6. The dotted black line is a smoothed background fit. The spectra as well as the background are derived from the fit provided by the PyMCA program. The doublets are *K*_*α*_ and *K*_*β*_ fluorescent radiation. The summed count rate above the black background line is shown in the top of each panel.

The fluorescent spectrum from mask 5, summed over all pixels, is shown in the three panels of Figure 7 for S, K and Fe, respectively. The analysis resulting from PyMCA splits the measured count rate into a fitted background (black dotted line) and the *K*_*α*_ − *K*_*β*_ doublets. The summed count rates of the doublets are also shown. The approximately spherical shape of the cell means that the somewhat tedious segmentation of cryo-SXT data to find the average path length $\langle {t}_{RBCcyt}\rangle$ can be avoided because it is just a fraction of the sphere diameter, the fraction being determined by the ratio of side length of the mask 5 to the diameter. The densities of S, K and Fe then follow from equation 2 and are shown in Table 1. With the volume of the sphere being around 80 μm^3^ one finds the total number of atoms in the RBC as given in line 8 of Table 1.

The experimentally derived molar ratio of S:Fe is very close to 3:1 as expected for Fe coordinated to heme in hemoglobin, both in the ice outside the cell and within the cell. Furthermore, the total number of Fe atoms in the RBC is close to one billion, in agreement with the expected amount of hemoglobin.

## Infected red blood cell in the schizont stage

Next we discuss a red blood cell (RBC) infected with the parasite in the schizont stage. Figure 2 shows the Fe, S and K color-maps at one projection angle, together with soft X-ray transmission at the same angle in grey scale. Interestingly, we find that the K ions are depleted in the RBC outside the parasite. The depletion of K in the RBC cytosol outside the parasite is in qualitative accordance with the review paper by Kirk^11^.

Figure 3 defines masks where the beam penetrates ice only (masks 1–4), ice and RBC cytosol (mask 5), ice and RBC cytosol and parasite cytosol (mask 6), and finally mask 7 penetrating in addition the digestive vacuole. The analysis proceeds then in subsequent steps by first using equation 1 to find the densities in the vitrified ice. The next step yields the densities in the RBC cytosol from mask 5 using equation 3 together with the ice densities found in step 1. Step 3 uses mask 6 for densities in the parasite cytosol using the now known densities in the RBC cytosol and ice, and finally step 4 uses mask 7 for the digestive vacuole. The results are shown in Table 1.

### Overlaying data from cryo-SXT and XF maps

Imagine a cubic volume around a red blood cell (RBC) as sub-divided into voxels, each given by a set of integer indices $(i,j,k)$. One can choose the orientation of the voxels, so the plane at constant *k* is perpendicular to the incident beam, implying that fluorescent maps show the sum of intensities along *k* for a chosen $(i,j)$, *cf*. Figure 5.

The so-called segmentation, obtained from the soft X-ray tomography, associates each voxel $(i,j,k)$ with a certain compartment, for example the digestive vacuole (DV), the entire parasite (P), the entire cell (RBC) or outside the cell (O). Each of these compartments, for example the parasite, is then given by a three-dimensional, binary matrix, ${M}^{P}(i,j,k)$, where an element is 1 if $(i,j,k)$ belongs to the parasite, and 0 otherwise. One can then overlay the fluorescent map ${C}^{Z}(i,j)$ with the parasite by an additional plotting of a chosen color at $(i,j)$, but only plotting if ${\sum }_{k}{M}^{P}(i,j,k) > 0$. Clearly, a binary matrix for the RBC cytosol is obtained by subtraction ${M}^{cytoRBC}(i,j,k)={M}^{RBC}(i,j,k)-{M}^{P}(i,j,k)$, and one can thus overlay the RBC cytosol with the fluorescent map by plotting a color in cell $(i,j)$ if ${\sum }_{k}{M}^{cytoRBC}(i,j,k) > 0$. Examples are shown in Figure 3. Note that the sum is the chord length ${t}_{cytoRBC}(i,j)$ of the RBC cytosol at that particular $(i,j)$ relative to the dimension of one voxel. There is one feature which the segmentation cannot account for, and that is the position of the RBC center along the beam direction within the vitrified ice sheet. For one given tilt angle there is therefore some uncertainty in determining the path length in ice of fluorescent radiation originating in the cell depending on whether the cell edge is close to or far from the downstream edge of the ice. This uncertainty is most significant for the softest fluorescent X-ray, namely that from sulfur. A solution to this ambiguity is to obtain data at two tilt angles 180° apart and require the same sulfur signal. This approach will be taken in future measurements.

## Discussion

The new aspect of our method is that it combines cryo soft X-ray tomography (cryo-SXT) with mapping of atomic elements in different compartments, in particular of Fe, S and K.

Focusing on hemoglobin digestion, we emphasize that S is present in the amino acids cysteine or methionine, whereas Fe is coordinated to the heme molecule. For a given cellular compartment, the question is whether these amino acids and the heme are together bound within hemoglobin. Alternatively, each could be part of other proteins. Moreover, heme can also be part of hemozoin (Hz) or free if it has just been released from hemoglobin (Hgb). The answer lies in the S to Fe ratio from X-ray fluorescence (XF) data.

Consider first the digestive vacuole. The Fe atoms of the heme can be bound in Hz crystals or in some protein, likely to be Hgb. By masking an area with no Hz crystals in projection, one would be able to conclude from the S to Fe ratio in the fluorescent spectrum from that mask whether the dominant protein in the digestive vacuole (DV) cytosol indeed is Hgb. The orientation of the infected red blood cell in this study did not allow such a mask where the beam would only irradiate the DV cytosol but not the Hz crystals. Therefore, the mask labeled 7 in Figure 3 will see the sum of Fe bound in Hz crystals and in the protein. Assuming that the dominant protein is Hgb, the number of Fe atoms in the cytosol would be 1/3 of the number of S atoms, or (10.4 · 10^7^)/3 = 3.5 · 10^7^ according to line 15 in Table 1. That means that out of a total of 39.7 · 10^7^ fluorescent Fe atoms, the rest of 36.2 · 10^7^ Fe atoms must be in the Hz crystals. A unit cell of Hz contains two Fe atoms and has a volume of 1.407 nm^3^, so the total volume of all Hz crystals should be 1.407/2 · 36.2 · 10^7^ nm^3^. This volume is indeed in accordance with the estimated value from the cryo-SXT data within an accuracy of 10%, consistent with the conjecture that Hgb is the dominant protein in the DV cytosol. In a separate experiment (submitted for publication) without S fluorescence but only Fe fluorescence it was possible to mask out a region in the DV free of Hz and therefore one could determine the Fe concentration of non-crystalline heme. This concentration indeed matches the one in the present sample of (26.0 · 10^6^)/3 from line 12 in Table 1 in the present sample. Next, consider the cytosol of the parasite, *cf* line 14 in Table 1. Here the ratio of S:Fe is around 40, very different from the hemoglobin ratio of 3, so the S atoms in the parasitic cytosol must be part of peptides incorporated into proteins other than hemoglobin, generated by the parasite.

Considering the whole cell, the total number of S atoms appears 20% lower than in the pristine red blood cell, so they must have ended up outside the infected red blood cell during earlier stages. In contrast, the total numbers of Fe atoms and K atoms are remarkably similar to those in a pristine cell. From the distribution of iron, we conclude that about 2/3 of the Hgb has been consumed by the parasite. Series of greyscale projections of cryo-SXT data show that the parasite in the infected red blood cell has three well developed nuclei, *cf* Figure 1. This corresponds to a schizont stage (stage classification according to Silamut *et al*.^10^). We are thus in position to compare our data to the data reported by Hanssen and co-workers^12^, in particular to their Figure 6 (see Table 2), and indeed both data sets agree.

**Table 2:** Comparison of volumes and hemoglobin concentration.

Source	Hanssen *et al*.^12^	This paper
Parasite volume (fL)	25 ± 5	28 ± 2
infected RBC volume (fL)	82 ± 15	97 ± 7
Hgb concentration (mM)	3.8 ± 0.5	3.2 ± 0.1

Finally, we focus on the concentration of potassium. The change in ion concentrations in the infected cells is essential for understanding how the cell maintains its volume^11^. The concentration of *K* ^+^ ions in the red blood cell cytosol (RBCcyto) from the pristine stage to the schizont stage has decreased by a factor of seven. This is in accordance with the review by Kirk^11^. However, all of the *K* ^+^ ions are maintained in the RBC, which is at variance with Kirk’s Figure 1 postulating that the *K* ^+^ ions are leaving the RBC by New Permeability Pathways (NPP)^11^. The K fluorescence is a useful marker for the assignment of compartments in addition to the cryo-SXT data.

We have shown that the combination of cryo soft X-ray absorption tomography (cryo-SXT) with cryo X-ray fluorescence (XF) imaging yields a reliable and precise method for determining the concentration of Fe, S and K in various compartments of a red blood cell infected by *Plasmodium Falciparum*. The ratio of concentrations of Fe and S determines whether the Fe is part of heme still coordinated to hemoglobin, or released therefrom, or part of hemozoin crystals. In addition the concentration of monovalent cations such as *K* ^+^ can be determined.

Both the cryo-SXT and the XF methods as described will be improved. For cryo-SXT, dual axis tomography using flat electron microscopy (EM) grids would reduce the missing wedge of our data therefore allowing higher precision in volume estimations of the different compartments and features in the RBC cryo-SXT data. For XF imaging the contaminating Cu radiation can be avoided by using aluminum EM grids. The accuracy of the self-absorption correction can be improved by establishing a 180° sample rotation so that upstream and downstream contributions can be separated. These improvements will enhance the measurements such that the atomic composition of smaller objects such as nuclei or liquid droplets may be obtained in spite of the relatively large volumes upstream and downstream from the object.

The biological perspective is to obtain images with cryo stabilization that preserves native structures for a series of stages at well-defined times after infection from the ring stages throughout the schizont stages. Such a movie of snapshots of the parasite development in the red blood cell, obtained in the wet stage without any specimen slicing or staining, should be most helpful in obtaining a reliable model and, subsequently, an intelligently designed drug preventing the asexual multiplication of the parasite in the red blood cell stage.

## Methods

### Sample preparation

Human red blood cells from voluntary healthy donor were supplied by the certified supplier Haema AG, Germany.

*Plasmodium falciparum* (3D7 strain) culture was grown in the lab in human erythrocytes at 5% hematocrit in HEPES-buffered RPMI 1640 medium containing Glutamax (Gibco), 10% human serum (Haema), 2% hypoxanthine, and 0.1 mg/ml gentamicin. Parasites were incubated at 37 °C in an atmosphere of 3% oxygen, 5% carbon dioxide, and 92% nitrogen. Late stage parasites were enriched by centrifugation at 1500 × g with 70% Percoll. A drop of 1–3 μl of the parasite culture was placed onto a 3 mm electron microscopy specimen grid made of copper and rapidly vitrified by high pressure freezing. The sample vitrification was conducted at the Institute of Integrative Neuroanatomy, Charite using Leica HPM100 setup.

### The Xray instruments

The X-ray source at the beamline ID16A at ESRF is a 28 mm period, in-vacuum undulator using the 3rd harmonic at 17.05 keV. The focusing of the beam down to a beam size of about 30 × 30 nm FWHM is provided by a KB set of aspherical, multilayer *W*/*BC*_4_ coated mirrors with a 1 per cent relative energy bandwidth at a distance of 185 m from the source. In the vertical plane the center of the mirror was 0.1 m from the image point so the geometrical demagnification was 0.1 m/185 m = 0.54 · 10^−3^. The relatively large horizontal electron beam size in ESRF necessitated a virtual beamspot 40 m downstream from the undulator and a resulting geometrical demagnification of 0.045 m/145 m = 0.31 · 10^−3^. The resulting intensity was around 2.5 · 10^10^ photons/sec. The X-ray fluorescence detector subtended a solid angle ΔΩ of 0.14. A typical scan in the $(i,j)$ plane comprised 150 × 150 pixels and took less than one hour using 0.1 sec per point.

The MISTRAL beam line at ALBA uses a bending magnet as source^18^. The Transmission X-ray Microscope (TXM, Zeiss, formerly X-radia) was set at an energy of 520 eV. A single-reflection elliptical glass capillary condenser focuses monochromatic light on to the sample, which is an EM grid at cryo-temperature. The transmitted signal is collected by an objective Fresnel Zone plate of 25 nm outermost zone width, and a magnified image is delivered to a direct illumination CCD camera. The spatial resolution in 2D is ~26 nm half pitch^19^ for tomography using a tilt range from −65 to 60 degree.

### PyMCA

The PyMCA software package^16^ calculates the fluorescent spectrum for a fitted amount of irradiated fluorescent atoms. The spectrum includes K-lines, L-lines and M-lines as well as escape lines from the Si detector, all broadened by the detector resolution. It accounts for detector pile-up and includes a fitted smooth background as well as scattering, shown as the thin black line in Figure 4D. The input is the atoms one expect to contribute to the spectrum, and for that we used Al, P, Si, S, K, Ca, Cl, Co, Ni, Cr, Cu, Br and Au. The output are the fitted lines for all these atoms. The examples are shown in Figure 7.
